# From big associations to big practices—Why normative modeling should be the default in personality neuroscience

**DOI:** 10.3389/fpsyg.2025.1701166

**Published:** 2025-11-04

**Authors:** Peiqian Wu, Yudie Chang

**Affiliations:** School of Educational Science, Anhui Normal University, Wuhu, Anhui, China

**Keywords:** personality neuroscience, small effect sizes, heterogeneity, normative modeling, brain-wide association, reproducibility, big data

## Introduction

Early studies reported enticing correlations between Big Five personality traits and brain structure (DeYoung et al., 2010; Kanai and Rees, 2011). Such findings supported the view that stable traits have identifiable neural substrates. However, over a decade of research revealed a more complicated picture: many reported brain-trait associations failed to replicate (e.g., Boekel et al., 2015), and the field struggled with so-called “voodoo correlations,” in which extremely high correlations were likely spurious (Vul et al., 2009). Subsequent critiques underscored that typical sample sizes were underpowered to detect the small effect sizes realistic for personality–brain links (Button et al., 2013; Dubois and Adolphs, 2016).

## Small effects and the need for big data

**Figure 1:**
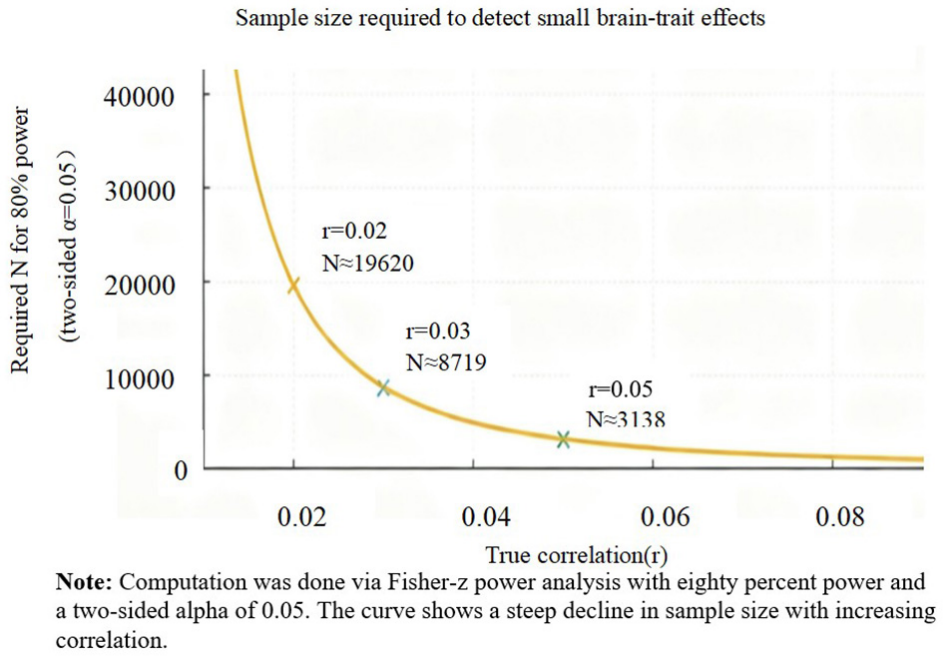
Sample size required to detect small brain–trait effects (80% power, two-sided α = 0.05). Markers indicate *r* = 0.02/0.03/0.05. Computed via Fisher-z power analysis.

Comprehensive investigations have found little to no evidence of robust brain–personality associations in large samples. For instance, Avinun et al. (2020) examined 1,107 individuals and reported no significant relationships between Big Five traits and multiple measures of brain morphometry. A systematic review and meta-analysis reached a similar conclusion, summarizing that there are no replicable structural brain differences as a function of Big Five traits (Chen and Canli, 2022). These results suggest that true associations, if present, are extremely small in magnitude. Power analyses and empirical work converge on the same message: detecting such tiny effects requires very large samples. Marek et al. (2022) showed that typical brain-wide association studies require thousands of subjects for reproducibility. Figure 1 illustrates this relationship: as effect size decreases, the sample size needed for 80% statistical power rises steeply. In practice, most historical studies in personality neuroscience were far too small, yielding underpowered analyses and spurious positives (Yarkoni, 2009; Button et al., 2013).

## Heterogeneity and the case for normative models

Heterogeneity is a key reason why averaging can obscure meaningful effects: individuals with the same trait score can show different neural patterns, and similar brain measures can accompany different trait expressions. Sex-dependent or subgroup-specific associations have been observed (Nostro et al., 2017), and idiosyncratic variation is the rule rather than the exception. Normative modeling provides a principled solution by estimating the expected distribution of brain measures given covariates (e.g., age, sex) and then characterizing each person by their deviation from that norm (Marquand et al., 2016, 2019). Instead of asking whether trait X correlates with region Y on average, we ask whether individuals with extreme trait values show atypical deviations relative to peers. This person-centered approach has already improved sensitivity in clinical domains, revealing heterogeneous deviation patterns in disorders such as schizophrenia and bipolar disorder (Wolfers et al., 2018). Resources like the Brain Charts for the human lifespan demonstrate how large multi-cohort datasets can be used to build robust normative references (Bethlehem et al., 2022).

## From big associations to big practices

Adopting normative modeling as a default approach represents a shift from chasing elusive average effects to embracing big practices—robust analytical habits that match problem complexity. Practically, this entails: assembling adequately powered samples via collaboration and data sharing; deriving individual deviation scores for relevant brain measures; testing preregistered, out-of-sample hypotheses about how deviations relate to personality; and reporting full model performance and failures. This agenda pairs naturally with prediction-oriented analysis (Yarkoni and Westfall, 2017), multivariate methods, and open science. It aligns with the idea that personality is about what makes individuals unique—and our methods should quantify that uniqueness instead of averaging it away.

## Conclusion

The era of small-N brain–personality correlation hunting is ending. By defaulting to normative modeling, personality neuroscience can better accommodate small effects and heterogeneity, leverage large datasets to establish meaningful baselines, and identify how and why certain individuals or subgroups deviate. Coupled with adequately powered designs and transparent, predictive workflows, this shift from big associations to big practices promises findings that are more robust, generalizable, and practically meaningful.
